# MATtrack: A MATLAB-Based Quantitative Image Analysis Platform for Investigating Real-Time Photo-Converted Fluorescent Signals in Live Cells

**DOI:** 10.1371/journal.pone.0140209

**Published:** 2015-10-20

**Authors:** Jane Courtney, Elena Woods, Dimitri Scholz, William W. Hall, Virginie W. Gautier

**Affiliations:** 1 Dublin Institute of Technology, Kevin St, Dublin, Ireland; 2 UCD Centre for Research in Infectious Diseases, School of Medicine and Medical Science, University College Dublin (UCD), Dublin, Ireland; 3 UCD Conway Institute of Biomolecular & Biomedical Research, School of Medicine and Biomedical Science University College Dublin (UCD), Dublin, Ireland; University of Birmingham, UNITED KINGDOM

## Abstract

We introduce here MATtrack, an open source MATLAB-based computational platform developed to process multi-Tiff files produced by a photo-conversion time lapse protocol for live cell fluorescent microscopy. MATtrack automatically performs a series of steps required for image processing, including extraction and import of numerical values from Multi-Tiff files, red/green image classification using gating parameters, noise filtering, background extraction, contrast stretching and temporal smoothing. MATtrack also integrates a series of algorithms for quantitative image analysis enabling the construction of mean and standard deviation images, clustering and classification of subcellular regions and injection point approximation. In addition, MATtrack features a simple user interface, which enables monitoring of Fluorescent Signal Intensity in multiple Regions of Interest, over time. The latter encapsulates a region growing method to automatically delineate the contours of Regions of Interest selected by the user, and performs background and regional Average Fluorescence Tracking, and automatic plotting. Finally, MATtrack computes convenient visualization and exploration tools including a migration map, which provides an overview of the protein intracellular trajectories and accumulation areas. In conclusion, MATtrack is an open source MATLAB-based software package tailored to facilitate the analysis and visualization of large data files derived from real-time live cell fluorescent microscopy using photoconvertible proteins. It is flexible, user friendly, compatible with Windows, Mac, and Linux, and a wide range of data acquisition software. MATtrack is freely available for download at eleceng.dit.ie/courtney/MATtrack.zip.

## Introduction

Optical highlighters comprise a class of fluorescent proteins which either turn on (Photo-Activation, PA) or change (Photo-Conversion, PC) their emission wave length in response to photo-stimulation with Ultra-Violet light [1,2]. Among the most popular are the monomeric *Anthozoa* derived Green-to-Red photo-convertible proteins (mEOS2, Dendra2 and mKikGR), which irreversibly photo-convert from a green to red fluorescent state upon irradiation with UV light [3,4,5,6,7,8]. This property has afforded biologists the ability to selectively label sub-populations of tagged-proteins and to track their sub-cellular migrations in real-time, significantly enhancing the understanding of complex biological processes [9,10,11,12,13].

A typical PC experiment consists of defining a Region of Interest (ROI) in the green channel and photo-converting the ROI to red using a short laser pulse. The movement of the PC protein is then monitored by time-lapse microscopy, revealing novel protein trafficking destinations and migratory patterns [9]. Typical analysis of PC data requires the extraction of fluorescence intensity values within the ROIs, widely handled by commercial microscope software control packages in conjunction with the open source project, ImageJ [14], and its associated plugins before using spreadsheet software to manually normalize and plot intensity values from different ROIs [15,16,17]. Nevertheless, this process can be very time consuming and prone to error, prompting a demand for a new software enabling the automated analysis of PC datasets [18]. While software packages are readily available for Fluorescence Recovery After Photobleaching (FRAP) datasets (e.g. Virtual FRAP, easyFRAP, FRAPCalc [19]), key experimental differences between FRAP and PC protocols (e.g. one color vs. two color time lapse microscopy) make these packages ill-suited for analysis of PC datasets [20]. In particular, PC experiments employ dual color time-lapse protocols in order to track the migration of a newly generated PC signal throughout the entire cell. As such, tracking of the PC signal relies upon appropriate extraction of signal information from two channels, as well as efficient normalization and quantification of fluorescent signals within multiple ROIs simultaneously. Increasingly, PC proteins are applied to investigate the dynamics of proteins residing in a-membranous cellular organelles (e.g. Nucleoli) or transient supra-molecular assemblies (e.g. Splicing Speckles or Stress Granules). However a poor Signal to Noise Ratio (SNR) can mask valuable information on protein residency and migration in these small cellular sub-compartments, as the fluorescent molecules undergoing PC include only a limited proportion of the total cellular population [9,21]. Issues also arise when handling large volumes of 2D images generated from live cell imaging studies, and which contain rapid changes in protein dynamics [22]. Hence, a more dedicated analysis package with tailored noise filtering and segmentation algorithms is required in order to successfully quantify and retain the low intensity, high frequency fluorescent signals obtained from PC experiments.

Here, we provide users with a new convenient toolkit, which can be easily incorporated into the image analysis workflow and significantly accelerates the process of determining trafficking patterns of Green-to-Red photo-convertible fusion proteins. We introduce MATtrack, a quantitative analytical tool, which is tailored towards processing datasets obtained from dual-color, multi-dimensional (x,y,t) live cell imaging studies using photo-convertible proteins, and which was developed in the technical computing language, MATLAB. Importantly, MATtrack comprises a simple user interface and its implementation requires no specialist programming knowledge.

## Materials and Methods

### Plasmids

pDendra2-C and pDendra2-Fibrillarin expression vectors were a generous gift from Dr. Konstantin Lukyanov (Institute of Bioorganic Chemistry, Russian Academy of Sciences, Moscow, Russia) [4]. Dendra2UBC9 was obtained by PCR amplification of the cDNA encoding UBC9 from pCDNA3-UBC9-SV5, obtained from Dr. Ronald Hay (University of St. Andrews, St. Andrews, UK) and cloning in frame into the EcoR1 sites of the Dendra2-C expression vector.

### Cell Culture

HeLa cells (ATCC CCL-2) were maintained in Dulbecco’s Modified Eagles Medium (DMEM) supplemented with 10% Foetal Calf Serum (FCS) at 37°C in a humidified atmosphere of 5% CO_2_. 24 hours before imaging, HeLa cells grown in μ-slides (IBIDI) were transfected with 50 ng of Dendra2, Dendra2Fibrillarin or Dendra2UBC9 complexed with 0.15 μl Fugene HD (Roche). Prior to photo-conversion experiments, DMEM was exchanged for HEPES buffered DMEM without phenol red supplemented with 10% FCS. [10].

### Image Acquisition

Image acquisition was carried out as described previously [23]. Briefly, HeLa cells transiently expressing Dendra2 fusion proteins (Dendra2, Dendra2-Fibrillarin and Dendra2UBC9) were imaged on a Nikon Eclipse Ti E Spinning Disk Confocal Microscope, at 37°C in a humidified atmosphere. Transfected cells were identified by exciting the Dendra2 signal (Green) with a 488nm laser at 1–8.5% laser power, and detecting emission at 512/518 nm. Next, a sub-cellular region was targeted using a FRAP-PA unit (Andor) and photo-converted with a short (1000μs/ pixel) pulse of a 405nm diode laser administered at 25% laser power. Photo-converted Dendra2 (Red) signal was obtain with a 561 nm diode laser at 25% laser power, and emission was detected at 624/40 nm. Protein trafficking events were monitored by recording a time series in the red channel. The MATtrack software, including data pre-processing, image analysis and the user interface, were implemented in MATLAB ver 7 (R14) but is compatible with more recent versions and has been tested up to MATLAB 2014b with Image Processing Toolbox.

### Image Analysis in Andor IQ2

Comparative analysis of Dendra2 protein trafficking was performed using AndorIQ2 software (Andor). The mean fluorescence intensity was quantified in user defined ROIs and exported to excel for further analysis. The data was normalized by subtracting the background signal and subsequently quantified over time. Movies of Dendra2 protein trafficking were generated using IMARIS (Bitplane).

## Results and Discussion

**Fig 1:**
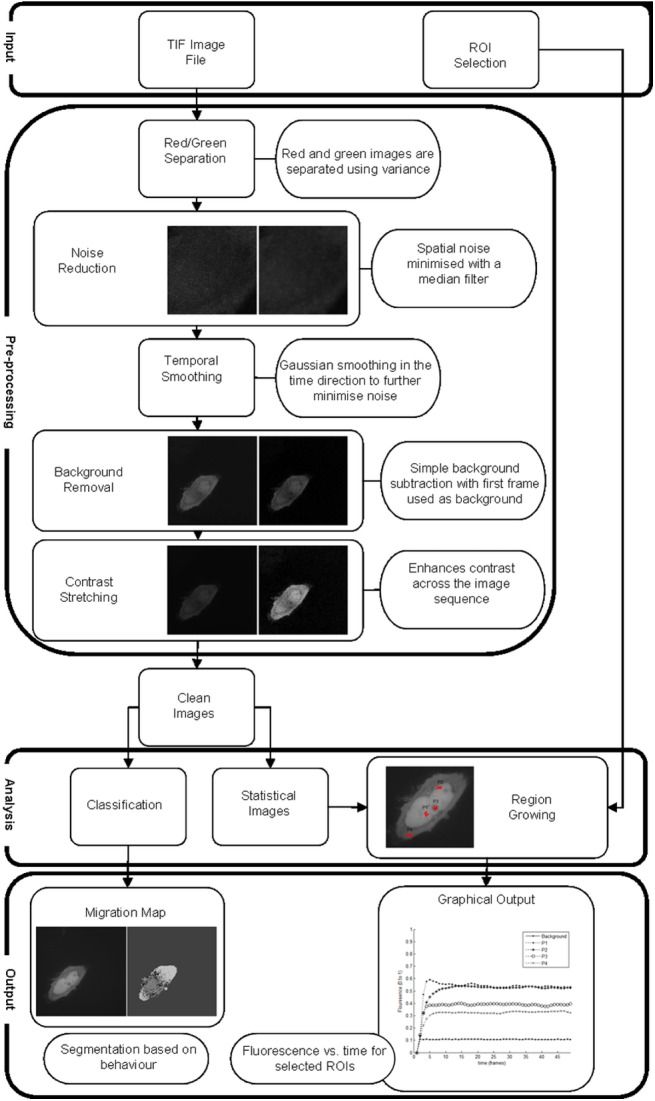
Overview of the image analysis workflow. The data set is exported as a Multi-Tiff file and analyzed using an algorithm developed in MATLAB. **Image processing:** The processing stage consists of green/red image separation, spatial and temporal noise filtering, background normalization and contrast enhancement. **Image analysis:** A migration map is generated automatically by classifying the signal variation of individual pixels over the time series. The user selects the location of the ROIs (a single mouse click for each), the regions are automatically created from these seed points and a graph of mean fluorescence of each ROI over time is automatically produced.

An overview of the MATtrack image analysis workflow is provided in Fig 1. MATtrack contains multiple processing algorithms to firstly separate a dual color time-lapse multi-tiff data set into its red and green components, before subjecting the red images to noise filtering, normalization, contrast stretching and temporal smoothing in order to improve detection of the PC signal. Next, MATtrack employs a Seeded Region Growing Algorithm to accurately delineate user selected sub-cellular ROIs, and subsequently detects protein trafficking events by automatically quantifying time-dependent variation in fluorescence intensity within the selected regions. Finally, MATtrack builds a “Migration Map” from a statistical model of the data set, providing a convenient means to visualize protein trafficking patterns and areas of significant accumulation.

We tested MATtrack using image sequences derived from PC time-lapse experiments of HeLa cells transiently expressing different Dendra2-fusion proteins. Dendra2 alone as well as Dendra2 fusion proteins consisting of the nucleolar protein Fibrillarin (Dendra2-Fibrllarin) or the SUMO-conjugating enzyme UBC9 (Dendra2UBC9) were employed to determine whether our image processing and analytical algorithms could discriminate between purely diffusive as well as directed trafficking behaviors, mediated by Nuclear Localization Sequences (NLS), protein-protein interactions or RNA binding motifs [24,25,26].

### Image Processing

#### Red/green image separation

Color information from dual color time-lapse protocols may not always be retained in the output file type (.tif). Consequently, the red and green component images in the image sequence require automatic separation. Here, the variance in the image is used to systematically distinguish between red and green images. Indeed, green images in the dataset capture the pre-PC signal from Dendra2 and contain more data and consistently higher variance than red images, even when a PC event has occurred prior to implementation of the acquisition protocol [27]. Hence, MATtrack compares the variance of fluorescence in the initial red frame, which represents the pre-conversion signal, with the variance of fluorescence in the initial green frame, which contains the full data. Since the pre-conversion state contains little or no signal in the red frame, this should have a very low variance while the green image will be very varied. Thus, MATtrack can clearly distinguish and separate the red and green frames.

#### Noise filtering

Images in the test data sets were acquired using a Spinning Disk Confocal Microscope (SDCM), generating an image sequence of high spatial and temporal resolution containing a low intensity, high frequency PC signal of interest. Potentially, this signal could be obscured by noise introduced during image capture or subsequent digitization and thus requiring a dedicated noise reduction algorithm in order to preserve the signal of interest.

Noise is identified by examining the images in both the spatial domain and the time direction, where sharp spatial variations in local neighborhoods as well as sharp local temporal changes (i.e. frame-to-frame) were classified as noise. Comparatively individual elements of the image should remain relatively homogeneous, while true temporal changes in signal should be relatively smoother and maintained over several frames.

**Fig 2:**
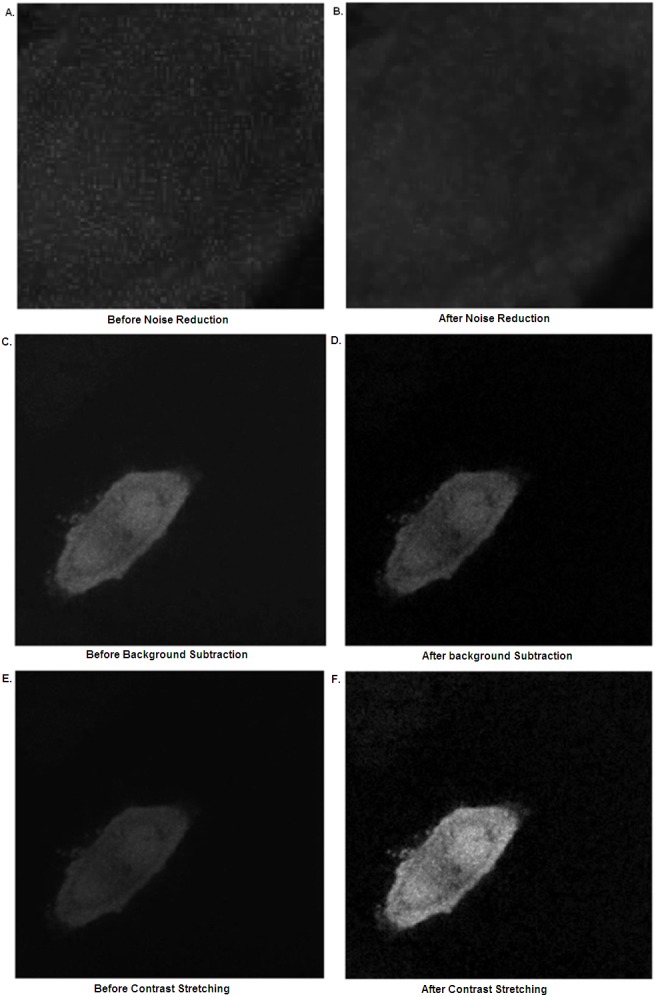
Image processing. A zoomed confocal image of a HeLa cell transiently expressing photo-converted Dendra2 (A) before and (B) after application of a median filter; (C) before and (D) after subtraction of the pre-conversion red image (background); (E) before and (F) after application of the Contrast Stretching Transform.

A 3x3 median filter, which examines the intensities in the local neighborhood of each pixel and sets the new pixel value to be the median (centre value) of the neighbors, is applied to reduce spatial noise and preserve image features without attenuating the high frequency signal of interest (Fig 2A–2F) [28,29].

The data set is then temporally smoothed by applying a Gaussian filter in the time direction, which sets each new pixel value to a weighted sum of its neighbors, thereby minimizing local frame-to-frame variation. A Gaussian filter was defined in the time (t) direction according to Eq 1:

$$G(t)=\frac{1}{\sqrt{2\pi }\sigma }e^{-\frac{t^{2}}{2\sigma^{2}}}$$ (1)

Where σ is the standard deviation of the filter. With σ = 1 and using a width of 5 (stretching across five frames), this subsequently translates to a 1-D mask with:

G=[0.0540.240.40.240.054]

This is passed across the image sequence and centered on each frame, transforming each pixel to the weighted sum of its temporal neighbors. Importantly, this smoothing is curtailed at the start and end of the image sequence, to prevent extension of the filter beyond the length of the image sequence.

#### Contrast Enhancement

An initial background extraction method was implemented in order to enable more accurate identification of ROIs during image analysis. Here, the first red frame in the image sequence (pre-conversion) is classified as background. This is subtracted from each subsequent frame, leaving the area of interest strongly contrasted with the background region (Fig 2C and 2D).

In order to maximize the contrast and increase the dynamic range of the image, the image sequence is contrast stretched and normalized, setting the maximum intensity in the whole sequence to 1 and the minimum to 0 (Fig 2E and 2F).

The maximum and minimum intensity values across the entire image sequence are determined (*I*_*min*_ and *I*_*max*,_ respectively) and the intensity of each pixel in the sequence, *I(x*,*y*,*t)* is scaled using the linear transform (Eq 2):

$$I\left(x,y,t\right)=\frac{I\left(x,y,t\right)-I_{\text{min}}}{I_{\text{max}}-I_{\text{min}}}$$ (2)

Of note, contrast stretching is applied across the entire sequence at once, as stretching frame by frame would lead to apparent fluorescence changes in the time domain.

### Image Analysis

#### Statistical Model

**Fig 3:**
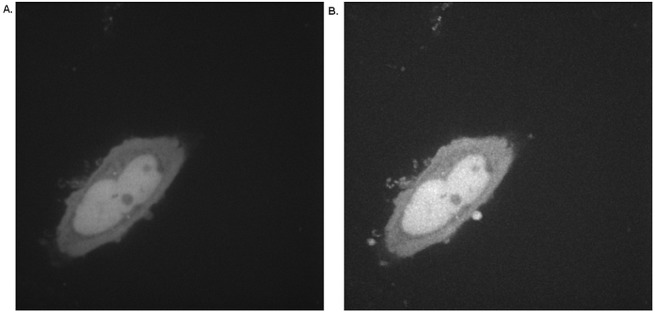
A statistical model of the image sequence. Construction of (A) the mean image **M** and (B) the standard deviation image **S** of a time series of a single HeLa cell transiently expressing photo-converted Dendra2.

Analytical protocols are implemented to identify sub-cellular destinations and map the trajectories of the protein of interest. A statistical model of the processed image sequence is built by calculating the mean image, **M**, and standard deviation image, **S**, determined from the mean, *M(x*,*y)*, and standard deviation, *S(x*,*y)*, at each pixel location across the entire image sequence (Eqs 3 and 4):

$$M(x,y)=\frac{1}{N}\sum_{t=1}^{N}I\left(x,y,t\right)$$ (3)

$$S(x,y)=\sqrt{\frac{1}{N}\sum_{t=1}^{N}{\left(I\left(x,y,t\right)-M\left(x,y\right)\right)}^{2}}$$ (4)

where *N* is the number of images in the sequence and *I(x*,*y*,*t)* is the intensity of each pixel. The mean image represents the average intensity of each pixel across the sequence, providing an improved quality image of the entire cell (Fig 3A), while the standard deviation image indicates which pixels exhibit high temporal variation, thus highlighting cellular areas of highest protein trafficking (Fig 3B).

#### Migration Map

To generate a map to visualize protein trafficking patterns and regions of accumulation, we designed a classification algorithm, which automatically examines the variation of each pixel during the image sequence. This approach was preferable to employing a machine learning algorithm as these systems require significant human intervention during the training phase, and the resultant systems can be unable to handle irregular images [30,31]. In contrast, the algorithm proposed here is advantageous as classification is performed by direct examination of the image sequence, requiring no human intervention in the classification and requiring no training, while allowing for adaptability to unusual images. Here, the region encompassing the cell is identified by locating the areas of highest variation (i.e. highest standard deviation). By analyzing the standard deviation image, **S**, and taking a threshold–defined as the mean of **S** plus one standard deviation of **S**–the cellular region is defined as:

$$A\left(x,y\right)=\{\begin{array}{l} 1\;\text{if}\;\text{S}\left(x,y\right)>\left(\bar{S}+\sigma_{S}\right)\\ 0\;\text{if}\;\text{S}\left(x,y\right)<\left(\bar{S}+\sigma_{S}\right) \end{array}$$ (5)

**Fig 4:**
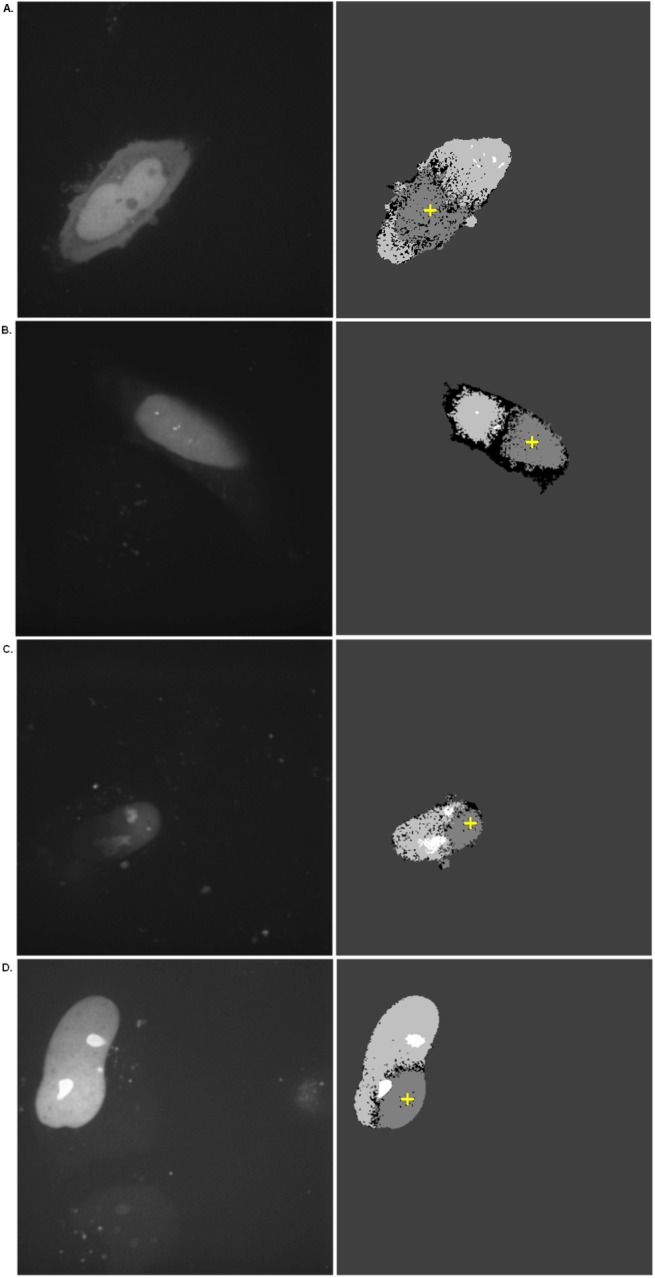
The Migration Map. (A) Dendra2; (B) Dendra2UBC9; (C) & (D) Dendra2-Fibrillarin. The sub-cellular migration of a selection of Dendra2 fusion proteins from the PC region is described in their mean (left) and corresponding classification images (right). The key is as follows: White: accumulation; Light grey: diffusion towards; Dark grey: injection; Darkest grey: background; Black: unclassified. The initial PC region is highlighted with a cross.

The region formed is then processed using morphological opening to remove spurious blobs [32]. Within this region, the pixels are then classified by observing the changes over time of each pixel in the region, generating a “Migration Map” of the photo-convertible protein intra-cellular movement throughout the time series (Fig 4A–4D). Here, if a cellular region is bright but dims as the image sequence progresses, it is classified as the original PC location. The centroid of this section is taken as the approximate PC point. Conversely–dim becoming bright–indicates a region to which the protein is migrating. Extreme brightness, either relative to the rest of the image or in absolute terms (i.e. saturation), suggests regions of high accumulation. Finally, regions that remain relatively dark with minimal variation are classified as background. (The unprocessed raw data is presented as S1 Movie and S2 Movie).

### Application

To test our classification algorithm, image sequences derived from PC time lapse experiments of HeLa cells transiently expressing different Dendra2-fusion proteins were analyzed (Fig 4A–4D).

Firstly, Dendra2 was employed as a model of diffusive behavior in a cellular environment, as it lacks interaction with cellular sub-compartments, and localizes throughout the nucleoplasm and cytoplasm (Fig 4A, left panel). After PC of a nucleoplasmic region, the corresponding region on the Migration Map is colored dark grey and the cytoplasm light grey, indicating diffusion of Dendra2 away from the PC region and across the nuclear envelope into the cytoplasm (Fig 4A, right panel). Of note, no white areas can be observed in the Migration Map, indicating Dendra2 does not preferentially accumulate in specific sub-compartments.

Next, the classification algorithm was examined to determine if it could accurately identify regions of protein accumulation using the fusion proteins Dendra2-Fibrillarin and Dendra2UBC9, which are known to accumulate in the nucleolus and PML nuclear bodies, respectively (Fig 4B–4D, left panel) [33,34,35]. In both case, a nucleoplasmic region was photo-converted and the migration map was used to describe the resulting trafficking behavior. Remarkably, the Migration Map correctly outlined how Dendra2-Fibrillarin or Dendra2UBC9 diffused through the nucleoplasm and specifically interacted with the nucleolus or PML nuclear bodies respectively (Fig 4B–4D, right panel; White regions). Hence, our algorithm correctly classified sites of protein accumulation even when they resided in very small sized sub-nuclear structures (PML nuclear bodies) which could be problematic for the user implementing manual ROI delineation alone

#### Region Selection

A simple user interface allows Regions of Interest (ROIs) to be selected and the corresponding fluorescent signal to be analyzed and outputted automatically in graphical format. Here, the user selects single points on the mean image, **M**, of the image sequence and a Seeded Region Growing Algorithm is used to define a ROI based on a set of corresponding nearby points. By automatically identifying a set of similar points, a regional average is calculated and tracked through the sequence. If only the selected point was tracked, this may cause issues as the selected point may be an outlier. The region selection also overcomes the issue of low signal to noise ratio, whereas an individually tracked pixel may vary dramatically due to noise.

**Fig 5:**
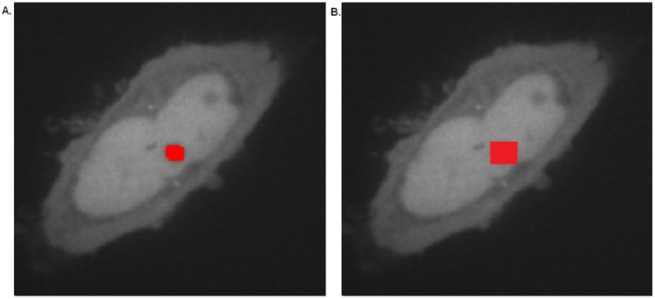
Comparison of automated selection methods for demarcating Regions of Interest (ROI) applied to the nucleoli. (A) Nucleolar ROI selection using a seeded region growing algorithm; (B) Nucleolar ROI selection using a fixed area around a selected point.

To define ROIs, we implemented a Seeded Region Growing Algorithm introduced by Adams & Bischoff [36]. In this technique, the user selected point is taken as a seed and its neighbors are searched to identify up to 50 local equivalent pixels, bypassing any outliers (Fig 5A). Here, equivalence is defined as Similar Mean Intensity, but alternative equivalence measures can be incorporated including: Standard Deviation, Region classification or distance from the selected point. Importantly, this algorithm abrogates the need for time-consuming manual delineation of the ROI. Furthermore, the algorithm employed here enables more accurate determination of ROIs when compared to other ROI selection algorithms where the ROI is defined as a fixed area around the selected point [37]. A comparison of the Seeded Region Growing algorithm with a standard ROI selection algorithm is shown in Fig 5. Here, selection of a small distinct nuclear region, such as a nucleolus occasioned the incorporation of unwanted nucleoplasm in the ROI, potentially resulting in erroneous quantification of the nucleolar signal (Fig 5B). In contrast, the Region Growing Algorithm employed here results in incorporation of pixels only belonging to the selected pixels region (e.g. cytoplasm, nucleoplasm, etc.) (Fig 5A).

The ROI selection method allows tracking of irregularly shaped areas which would make it compatible with other trafficking behavior including the trafficking of proteins associated with cytoplasmic membrane, the endoplasmic reticulum, the Golgi apparatus, endosomes, and lytic compartments. Nevertheless these remain to be tested and validated.

#### Graphical Output

**Fig 6:**
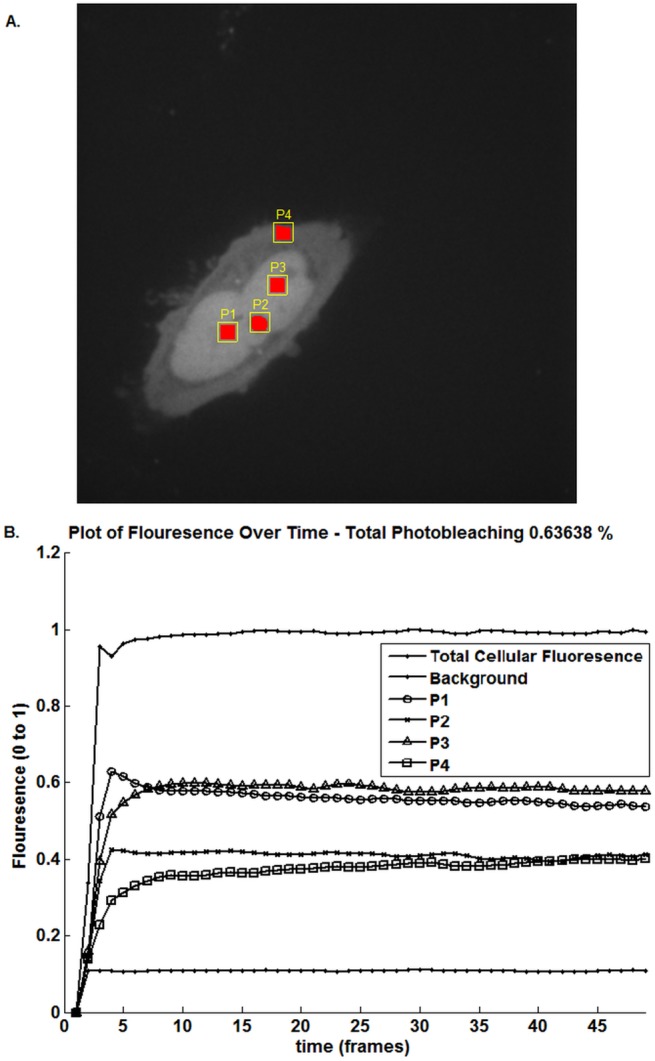
Automated analysis of Dendra2 sub-cellular trafficking in HeLa cells. (A) User defined cellular ROIs using the seeded region growing algorithm. P1 is the PC point; P2 is a distant nucleoplasm region; P3 is a nucleolus and P4 is a region of cytoplasm. (B) Quantification of photo-converted Dendra2 fluorescence in user defined ROIs over time.

After ROI selection, the average fluorescence intensity of the ROIs over the image sequence as well as the background are quantified and plotted against time providing an integrated view of protein re-localization to different cellular regions (Fig 6). For comparison the Dendra2 and Dendra2UBC9 datasets are provided as S1 Movie and S2 Movie respectively. This much-used method of identifying protein re-localization events by constructing a graph of mean fluorescence intensity over time from individual user-selected regions is seen in [38,39]. The user can also utilize the migration map to identify points or regions of interest based on their trafficking behavior. The size of the selected regions and number of image points can be specified by the user or will revert to pre-defined defaults (a maximum of ten points selected, with each region extending to a maximum of 250 pixels).

#### Accounting for Photobleaching

Because photo-bleaching can lead to erroneous trafficking behaviors in PC experiments, the total cell fluorescence must be examined to determine whether any reduction in fluorescence has occurred throughout the sequence. Any drop in fluorescence is reported as a percentage to the user so that low quality datasets may be discarded. Moreover, photobleaching is automatically corrected by normalizing the signal in the tracked ROIs against the total cellular fluorescence at each time point.

#### MATtrack validation compared to AndorIQ2: Tracking Dendra2UBC9 sub-cellular migration

**Fig 7:**
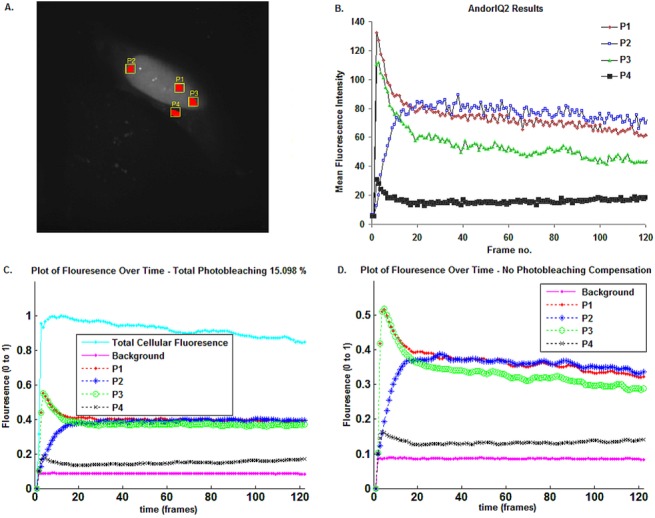
Comparison of Dendra2UBC9 protein trafficking obtained for both AndorIQ2 and MATtrack software. (A) The selected ROIs; (B) Dendra2UBC9 trafficking in HeLa cells analyzed using AndorIQ2; (C) The same sample analyzed using MATtrack; (D) Analysis using MATtrack with photobleaching compensation removed for direct comparison with AndorIQ2.

To validate our software accuracy in tracking PC protein re-localization events, we performed a comparative analysis of Dendra2UBC9 sub-cellular trafficking in HeLa cells using AndorIQ2 software (Fig 7). Here, the photo-converted nucleoplasmic region (P1), 2 additional nucleoplasmic regions (P2 and P3) and one additional cytoplasmic region (P4) were delineated manually using the SDCM acquisition software AndorIQ2, which automatically quantified the MFI in the defined ROIs over the time series. The data was then exported to excel, where the ROI MFI was normalized to the background by the user before being plotted against time (Fig 7B), which was completed in 10 minutes. In parallel, we employed automated analysis of the same ROIs using MATtrack, which was completed in approx. 2 minutes (Fig 7C). Moreover, MATtrack enabled automatic monitoring of the total photo-bleaching in the dataset, which was determined to be 15.098%. Comparison of the AndorIQ2 and MATtrack output results showed highly similar trafficking profiles. Dendra2UBC9 trafficked from the PC nucleoplasm and migrated to the other nucleoplasmic regions (P2 and P3). In parallel we observed a lower signal in the cytoplasm (P4), indicating a small proportion of Dendra2UBC9 was re-localizing to the cytoplasm. Close inspection revealed sharp frame-to-frame variation in signal processed using AndorIQ2, which had been removed by MATtrack’s integrated noise filtering algorithms. In addition, we observed a steady decline in Dendra2UBC9 signal in ROIs P1 and P2 in the AndorIQ2 dataset, which was absent from the MATtrack dataset. Potentially, this signal represented “true” Dendra2UBC9 trafficking away from these regions. Alternatively, this decline was an artifact of photo-bleaching. To distinguish between these possibilities, we re-analyzed the dataset in MATtrack without photo-bleaching correction (Fig 7D), resulting in a decrease in signal in ROIs P1 and P2 and indicating that photo-bleaching was responsible for the different outputs between the two methods. Importantly, this highlighted that AndorIQ2 and MATtrack performed comparably for analyzing PC protein trafficking, however the automatic integration of ROI selection, image processing and analytical algorithms in MATtrack resulted in significantly faster result output than AndorIQ2. We anticipate that MATtrack would extend the same advantages over other commercial software packages with integrated image acquisition and analytical protocols (Olympus, as well as the open source software ImageJ, which requires time consuming manual ROI delineation and data export to additional statistical software packages). Hence, MATtrack streamlines the image analysis workflow and enables more rapid dataset analysis compared to other software packages, increasing the rate of result output, giving the user a more comprehensive view of protein trafficking at the single cell level in a shorter time frame.

## Conclusion

MATtrack is a new software tool that automates the analysis of PC datasets in order to accurately monitor and characterize live protein trafficking *in vivo*. MATtrack implements multiple algorithms to process the raw data, alleviating the burden of identifying appropriate processing techniques which can pose significant challenges to the non-expert user [40]. MATtrack significantly streamlines the image analysis workflow to produce meaningful, quantitative results. Finally, its open-source code enables other programmers to refine existing algorithms and contribute new algorithms to expand MATtrack to suit their individual needs.
