# Correction: MATtrack: A MATLAB-Based Quantitative Image Analysis Platform for Investigating Real-Time Photo-Converted Fluorescent Signals in Live Cells

**DOI:** 10.1371/journal.pone.0143074

**Published:** 2015-11-11

**Authors:** The PLOS ONE Staff

The Supporting Information files S1 Movie, S2 Movie, S1 File and S2 File do not appear in the published article. The publisher apologizes for the error. Please view the Supporting Information files below.

## Supporting Information

S1 MOVIE Timelapse movie of the Dendra2 unprocessed raw data. Results from this data processed with MATtrack can be seen in Fig 6.
(MOV)

S2 MOVIE Timelapse movie of the Dendra2UBC9 unprocessed raw data. Results from this data processed with MATtrack and AndorIQ2 can be seen in Fig 7.
(MOV)

S1 File MATtrack Guide. Installation and operation instructions for MATtrack.
(PDF)

S2 File MATtrack. (ZIP)
