# Supplementary material for: Correction: MATtrack: A MATLAB-Based Quantitative Image Analysis Platform for Investigating Real-Time Photo-Converted Fluorescent Signals in Live Cells
Source: PLoS One. 2015 Nov 11;10(11):e0143074. doi: 10.1371/journal.pone.0143074 (PMC4641583; doi:10.1371/journal.pone.0143074)
Supplement: S1 File — Installation and operation instructions for MATtrack. (PDF) [file pone.0143074.s003.pdf]

MATtrack Installation and Operation

MATtrack is compatible with all versions of MATLAB with the standard Image Processing Toolbox. It is freely-available at eleceng.dit.ie/Courtney/MATtrack.zip and is included here as supporting information. The installation of MATtrack consists of unpacking the compressed folder, which contains a collection of MATLAB scripts (Classify, Run and Region) to the desired destination folder. To run MATtrack, the Run m-file must first be opened in MATLAB by selecting "*File*" and then "*Open*" in the menu bar and finally selecting *Run.m* from the target folder. Alternatively, it can be opened by double-clicking on it in the target folder. It is run by selecting *"Run"* from the editor's toolbar or by typing 'Run' in the MATLAB command line.

Data obtained through live cell PC experiments must be saved in the multi .tif format for import into MATtrack. On execution of the Run script, an *"Open File"* dialog box will open. This allows the user to find and select the .tif file of interest for processing.

After running the *Run.m* script and selecting the desired .tif file, the image processing (contained in *Run.m)* and analytical (found in *Region.m* and *Classify.m*) elements of the algorithm will now run automatically, generating the MATtrack user interface. This consists of the *"Classification"* panel, containing the Migration Map, and the *"Region"* panel, which contains the processed Mean image of the dataset. In the "*Region*" panel, the approximate PC point is marked as "*Injection*" on the Mean cell image. Instructions are given to the user (*"Select up to 9 points of interest by clicking on them. Press ENTER when finished"*) in order to generate a graph of Mean Fluorescence in each frame for each ROI (denoted as "P1-9") selected. The background signal is automatically graphed alongside the selections for comparison.
